# Influence of the knot position on the union of the greater trochanter after bipolar hip arthroplasty via the modified Dall approach: a prospective non-randomized study

**DOI:** 10.1186/s12891-021-04005-1

**Published:** 2021-02-10

**Authors:** Masanao Kataoka, Hiroshi Fujita, Hiroaki Hara, Hideto Harada, Yuki Okutani, Yoshiki Murotani

**Affiliations:** 1 Institute for Joint Replacement, Department of Orthopedic Surgery, Kyoto Katsura Hospital, Yamada-hirao-cho 17, Nishikyo-ku, Kyoto, 615-8256 Japan; 2 Department of Rehabilitation, Kyoto Katsura Hospital, Yamada-hirao-cho 17, Nishikyo-ku, Kyoto, 615-8256 Japan

**Keywords:** Bipolar hemiarthroplasty, Modified Dall approach, Knot position, Complications of the greater trochanter

## Abstract

**Background:**

In our institute, all elderly patients with displaced femoral neck fracture were treated with cemented bipolar hemiarthroplasty (BHA) using the modified Dall approach. To our knowledge, there are no reports on the knot position of the greater trochanter reattachment. The aim of this study was to determine influence of two knot positions (anterior or posterior) on the complications of the greater trochanter.

**Methods:**

This is a prospective non-randomized study conducted on 95 elderly patients (95 hips) from September 2013 to December 2017. The knot position was changed from anterior to posterior alternately. The X-ray images obtained immediately after the operation were compared with those obtained at 3 months postoperatively; thereafter, the status of the greater trochanter was classified into three types: type A, no apparent shifting and fracture; type C, over 1-mm shifting of the fragment; and type F, fracture of the greater trochanter.

**Results:**

Regarding age at operation, sex, BMI, size of the greater trochanteric fragment, stem type, and surgeon, there was no significant difference between two groups. In the anterior group, 34 hips (72.3%), 5 hips (10.6%), and 8 hips (17.0%) were classified under type A, C, and F, respectively. In the posterior group, 44 hips (91.7%), 1 hip (2.1%), and 3 hips (6.3%) were classified under type A, C, and F, respectively. There were significantly fewer greater trochanteric complications in the posterior group.

**Conclusions:**

The posterior knot position improved the union of the greater trochanter after BHA compared with the anterior knot position.

**Trial registration:**

We had approved IRB at our hospital clinical research review committee. Retrospectively registered.

## Background

According to the National Institute for Health and Care Excellence guidelines [1], replacement arthroplasty (total hip replacement or hemiarthroplasty) is recommended to patients with displaced intracapsular hip fracture.

There are three main categories of surgical approaches to hip arthroplasty: lateral approaches (LAs), posterior approaches (PAs), and anterior approaches (AAs). A recent meta-analysis [2] showed that PAs are associated with a higher risk of dislocation and re-operation than AAs and LAs. In our country LAs were relatively widely used and several good results were reported [3, 4].

In our institute, all elderly patients who had displaced femoral neck fracture were treated with cemented bipolar hemiarthroplasty (BHA) using the modified Dall approach [5], in which the greater trochanter was cut partially with the gluteus medius muscle. The modified Dall approach is a superior approach in that it can provide sufficient visualization during total hip arthroplasty (THA) or BHA. This approach can be used in cases requiring bone grafting because of acetabular dysplasia and can avoid excessive detachment of the soft tissues. However, this approach requires cutting apart of the greater trochanter. Therefore, it is necessary to take measures to prevent complications around the greater trochanter. Dislocation of the fragment may lead to non-union, and fragility of the posterior part of the greater trochanter may lead to fracture of the tip. In past procedures performed in our institute using the original Dall method, in which non-absorbable polyester sutures (Ethibond, Johnson & Johnson K.K., Tokyo, Japan) were passed through the greater trochanter, there were many complications observed. Two studies previously reported that ultra-high molecular weight polyethylene (UHMWPE) fiber cables yielded a sufficient degree of fixation equal to that in metal wires in spinal fusion surgery [6, 7]. Therefore, the fixation materials were changed from non-absorbable polyester sutures to UHMWPE fiber cables. The UHMWPE fiber cables were passed around the femur instead of through the greater trochanter, and the gluteus minimus tendon was partially cut apart, which enabled the reduction of the traction force against the greater trochanter. The trochanteric fragment was reattached at the original position using two UHMWPE fiber cables (3.0 mm in width) (NESPLON Cable System, Alfresa Pharma Co., Osaka, Japan). The cables were tightened around the femur using an anterior loop and were tied up using a double-loop sliding knot. However, there were some cases in which the fragments were displaced or fracture occurred at the greater trochanter.

We thought that knot position was important because knots caused local irritation and stimulated inflammation around the greater trochanter. To our knowledge, there was no reports on the knot position of the greater trochanter reattachment. There were two knot positions: anterior and posterior to the fragment. The aim of this study was to determine the influence of these two knot positions on the complications of the greater trochanter, and which knot position was clinically superior.

## Methods

**Fig. 1:**
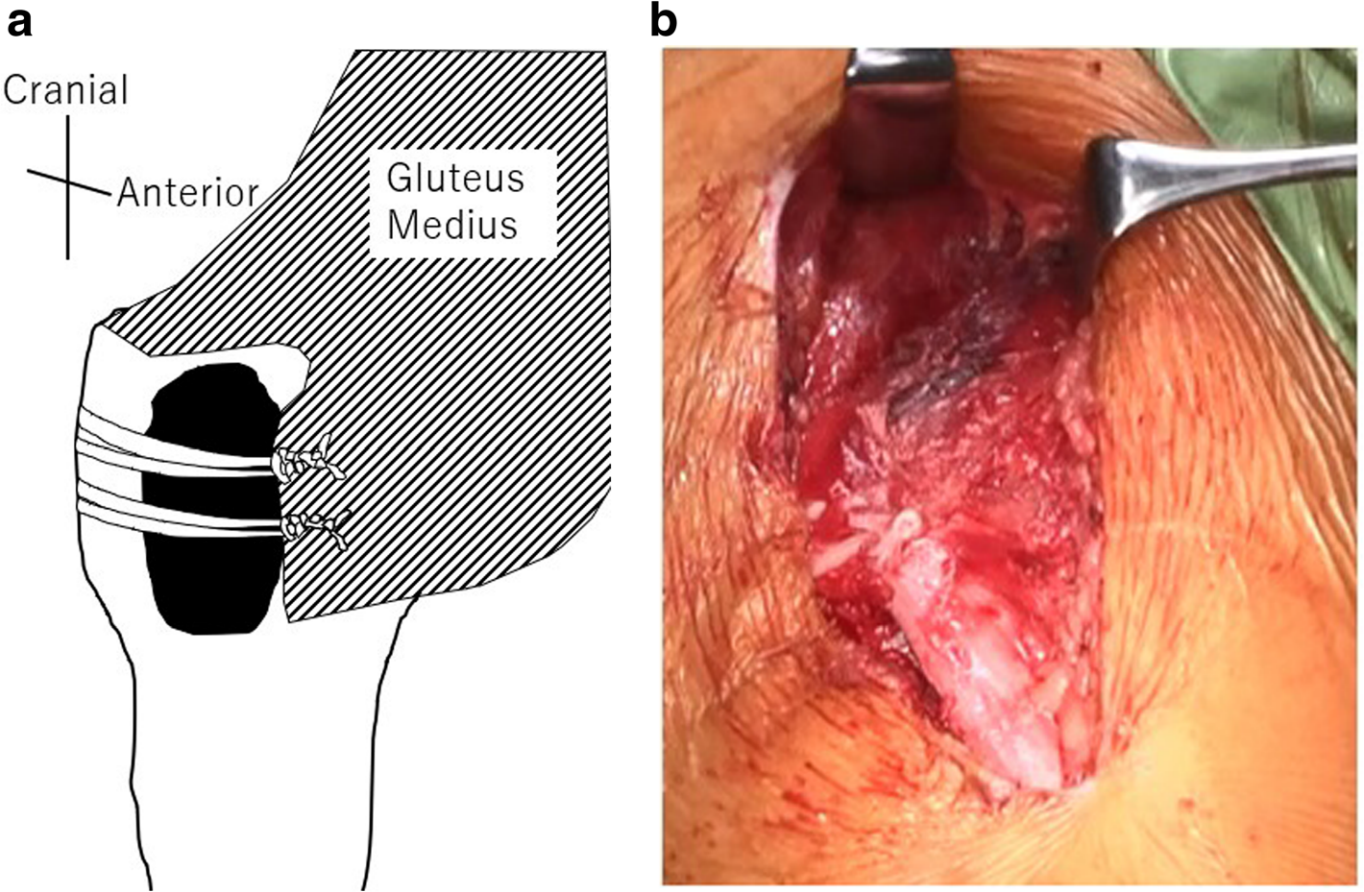
**a** is schema of the anterior knot position of the right femoral hip; **b** is an operative photograph of a case of anterior knot group

**Fig. 2:**
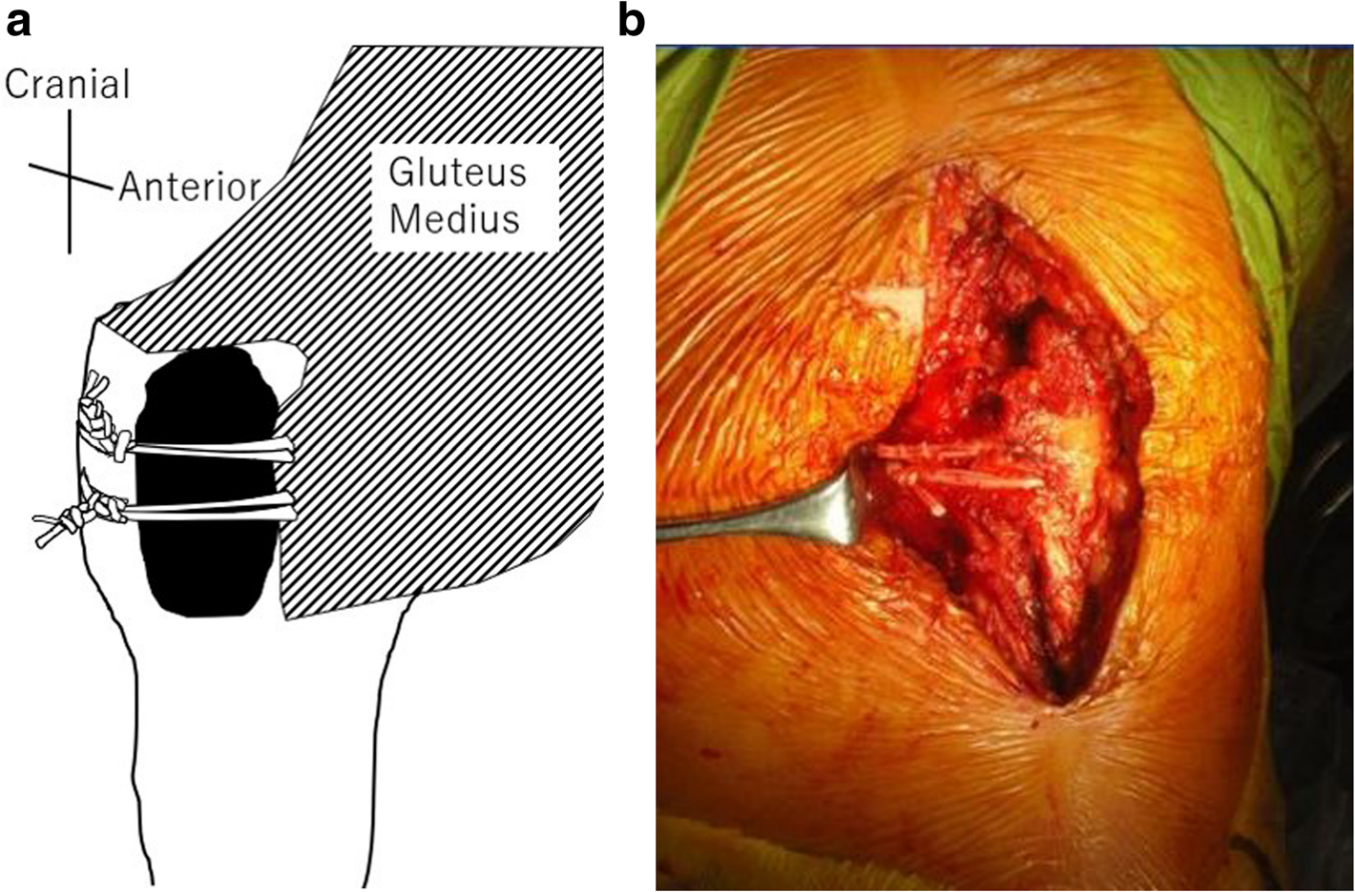
**a** is schema of the posterior knot position of the right femoral hip; **b** is an operative photograph of a case of posterior knot group

This study was a prospective non-randomized study conducted on 95 elderly patients (95 hips) with displaced femoral neck fracture who were treated with cemented BHA in our institute from September 2013 to December 2017. This study adhered to CONSORT guidelines. Sample size was determined that p1 = 0.3, p2 = 0.6, α = 0.05, 1-β = 0.8 resulted 42. Inclusion criteria were diagnosed of displaced femoral neck fracture, then 114 patients (114 hips) were met the criteria. Exclusion criteria were patients who had osteoarthritis or dysplasia (Sharp angle over 45 degrees) of the same side of fracture and patients who had no tolerance of surgery. Six patients (6 hips) met the former exclusion criteria, then they were treated with cemented THA. Thirteen patients (13 hips) met the latter exclusion criteria, then they were treated conservative. The knot position was changed from anterior (Fig. 1) to posterior (Fig. 2) alternately, and the surgeon did not know which knot position to adopt until just before the knotting. A third party other than the doctor or nurse assigned the knot position. The anterior position was used in 47 hips and the posterior position in 48 hips. This study was approved by our institutional review board, and Informed consent was obtained from each patient.

All operations were performed using the modified Dall approach. A chisel was used to cut the greater trochanter partially. The fragment thickness was maintained at 10 mm, and the fragment was not cut through entirely. In this manner, the osteotomy surface had a ramp, which prevented the fragment from shifting anteriorly. The fragment of the greater trochanter was measured using Vernier calipers: length (maximum cranio-caudal length), width (maximum antero-posterior length), and thickness before the fragment was reattached. The estimated volume of the fragment was calculated as follows: length × width × thickness.

In order to pull the fragment 15 mm posterior to the original position, the tendinous portion of the gluteus minimus was cut before reattaching. For the fixation method, two 3.0-mm-width UHMWPE fiber cables were pathed around the femur using anterior loops; thereafter, the cables were tied up using a double-loop sliding knot. The gun tensioning system (Tightning Gun TGL, Alfresa Pharma Co., Osaka, Japan) at more than 20 kg was used, and the cables were later tied four times by hand and finally tied once using the gun tensioning system at more than 20 kg.

Eleven Exeter stems (Stryker Orthopedics, Mahwah, New Jersey) and 84 SC stems (KYOCERA Medical, Osaka, Japan) were used. Exeter stem is the cemented polished double-tapered stem, and it has excellent outcomes in Japan [8, 9]. SC stem is the cemented polished triple-tapered stem. All stems were implanted with two packs of Simplex P bone cement (Stryker Orthopedics, Mahwah, New Jersey) and using the interface bioactive bone cement technique [10–12].

**Fig. 3:**
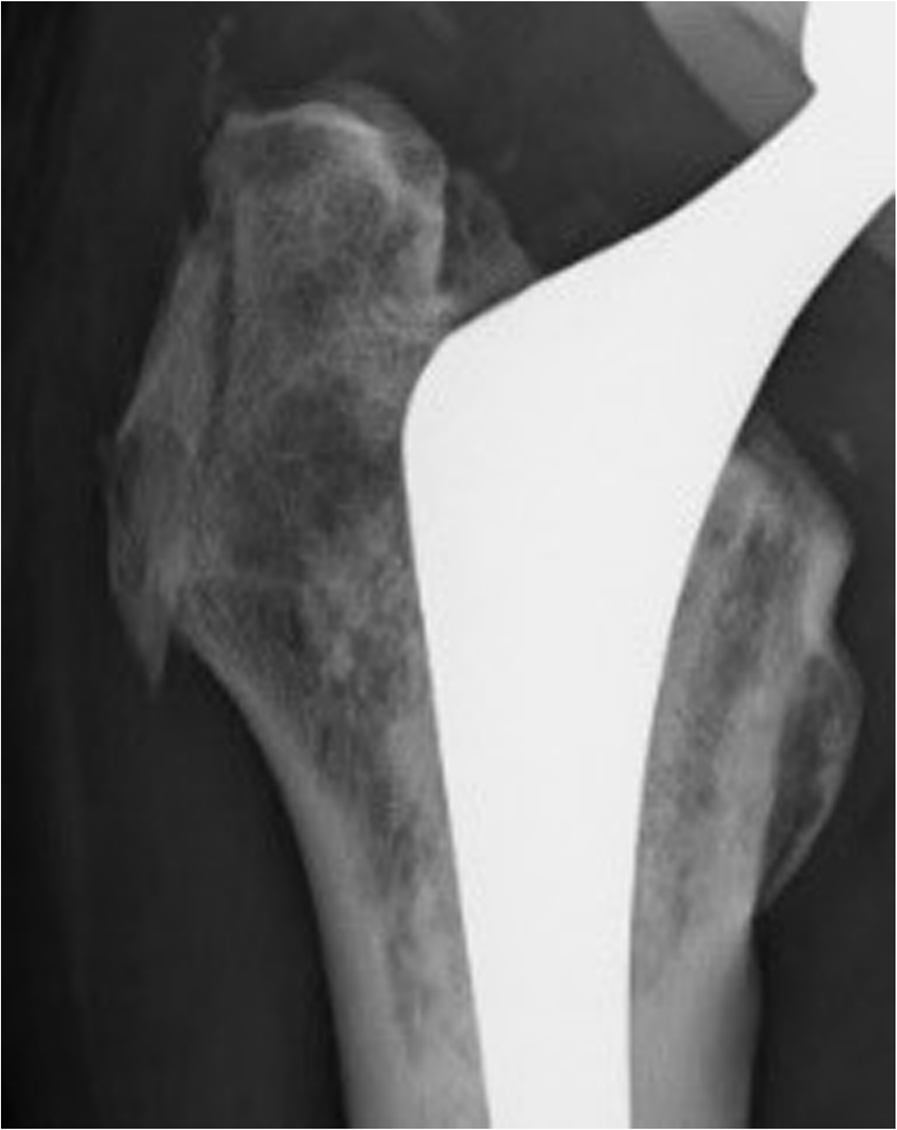
A case of type C. The trochanteric fragment was shifted distally over 1 mm at 3 months postoperatively

**Fig. 4:**
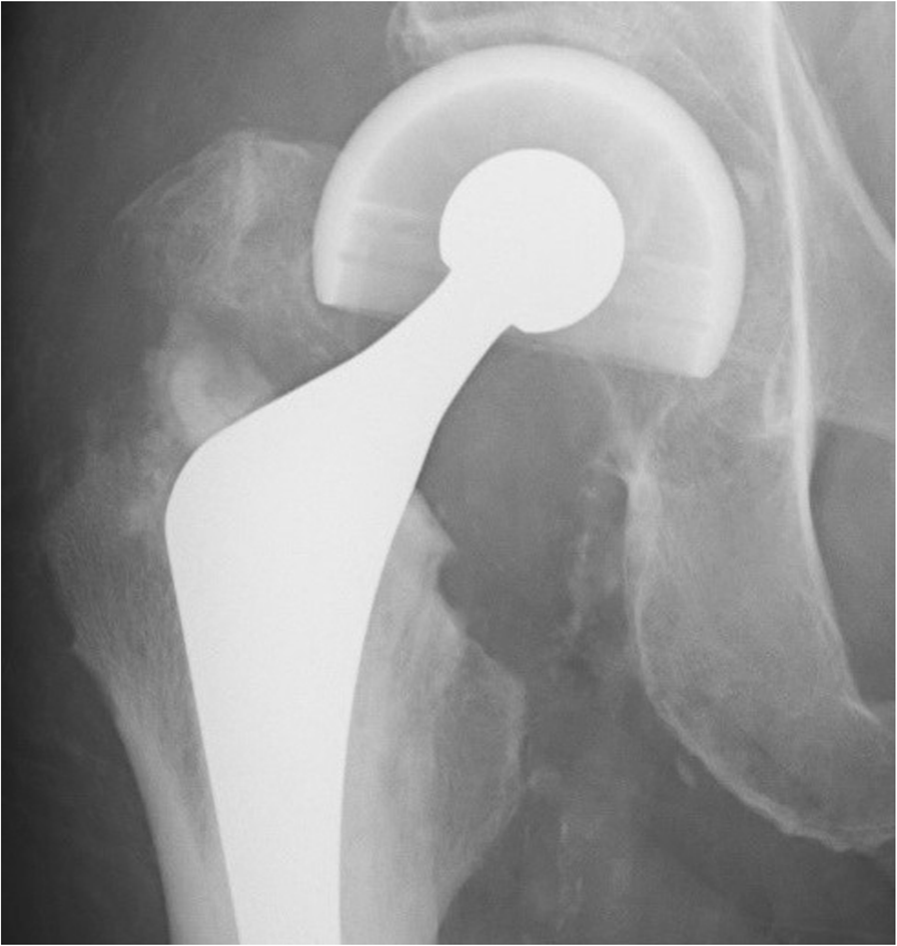
A case of type F. Fracture was occurred at the greater trochanter at 3 months postoperatively

The antero-posterior supine-positioned X-ray images obtained immediately after the operation were compared with those obtained at 3 months postoperatively, without informing the surgeons, patients, physical therapists, and radiologists which knot positions were applied. Osteotomy healing or union of the greater trochanter was then classified into three types: type A, no apparent shifting of the trochanteric fragment and fracture of the greater trochanter; type C (Fig. 3), over 1-mm shifting of the trochanteric fragment; and type F (Fig. 4), fracture of the greater trochanter. Three doctors (HH, MK, and HF) classified the status of the greater trochanter. Disagreements were resolved by consultation. The patients’ age at operation, sex, and body mass index (BMI); size of the greater trochanteric fragment; type of the stem; and experience of the surgeon (surgeon A: 20 years of experience, surgeon B: 15 years of experience; “others”, C: 5 years of experience and D: 15 years of experience) were analyzed using the t-test and chi-square test. C and D performed operations in fewer than five hips; thus, they were classified as “others”.

All analyses were performed using Statcel 4 (OMS Publ., Tokyo, Japan), which is an add-in software of Excel (Microsoft, Redmond, Washington). The statistical significance level was set at a value of < 5%.

## Results

Regarding age at operation, sex, BMI, size of the greater trochanteric fragment, estimated volume of the fragment, type of the stem, and surgeon, there was no significant difference between the anterior and posterior groups (Table 1).

**Table 1:** Patient characteristics

Parameter	Anterior group	Posterior group	*p* value
Number of hips	47	48	
Age at operation; years old (range)	82.4 ± 7.0 (69–95)	82.6 ± 7.9 (62–98)	0.89
Sex, male:female	14:33	18:30	0.43
Body mass index; kg/m2 (range)	20.1 ± 3.7 (13.4–27.7)	18.9 ± 3.4 (12.2–28.3)	0.10
Size of the greater trochanteric fragment			
length; mm (range)	33.2 ± 5.7 (23–45)	33.3 ± 5.3 (22–45)	0.93
width; mm (range)	21.7 ± 4.1 (14–34)	21.4 ± 4.0 (14–30)	0.76
thickness; mm (range)	8.9 ± 1.8 (5–14)	8.8 ± 1.9 (5–12)	0.81
estimated volume; mm3 (range)	6810 ± 3547 (1820–17,640)	6706 ± 3213 (2030–13,766)	0.89
Type of the stem			0.38
Exeter	4 (8.5%)	6 (12.5%)	
SC	43 (91.5%)	42 (87.5%)	
Surgeon			0.31
A	26 (55.3%)	30 (62.5%)	
B	16 (34.0%)	10 (20.8%)	
Others	5 (10.6%)	8 (16.7%)	
Type of the greater trochanter			0.045^*^
Type A	34 (72.3%)	44 (91.7%)	
Type C	5 (10.6%)	1 (2.1%)	
Type F	8 (17.0%)	3 (6.3%)	

mean ± standard deviation

^*^means less than 5%

In the anterior group, 34 hips (72.3%), 5 hips (10.6%), and 8 hips (17.0%) were classified under type A, type C, and type F, respectively. In the posterior group, 44 hips (91.7%), 1 hip (2.1%), and 3 hips (6.3%) were classified under type A, type C, and type F, respectively. A significant difference was found in type A between the two groups. A significant difference was also found in the BMI (*p* = 0.047) and suturing position (*p* = 0.013) between type A and type C + F (Table 2).

**Table 2:** Comparison between type A and C + F

Parameter	Type A	Type C + F	*p* value
Number of hips	78	17	
Age at operation; years old	82.7 ± 7.4	81.7 ± 7.5	0.60
Sex, male:female	28:50	4:13	0.25
Body mass index; kg/m2	19.2 ± 3.6	21.0 ± 3.2	0.047*
Size of the greater trochanteric fragment			
length; mm	33.2 ± 5.8	33.4 ± 4.2	0.89
width; mm	21.6 ± 4.0	21.3 ± 4.3	0.83
thickness; mm	8.9 ± 1.8	8.5 ± 2.0	0.36
Type of the stem			0.43
Exeter	9 (11.5%)	1 (5.9%)	
SC	69 (88.5%)	16 (94.1%)	
Surgeon			0.25
A	49 (62.8%)	7 (41.2%)	
B	19 (24.4%)	7 (41.2%)	
Others	10 (12.8%)	3 (17.6%)	
Knot position			0.013^*^
Anterior	34 (43.6%)	13 (76.5%)	
Posterior	44 (56.4%)	4 (23.5%)	

mean ± standard deviation

^*^means less than 5%

## Discussion

There are some reports of patients who had displaced femoral neck fracture and were treated with BHA via the modified Dall approach [13]; however, to our knowledge, there is no report on greater trochanteric complications and size of the greater trochanteric fragment. A hypothesis was that the fragment was larger, so as type F was increased, and the fragment was smaller, so as type C was increased. There was no statistically significant difference for complications with the size of the greater trochanteric fragment. However, knot position greatly influenced complications of the greater trochanter.

Oe et al. [3] reported that the incidence of greater trochanteric complications was 7.9% (37/466 hips) in their THA cases via the modified Dall approach using two UHMWPE fiber cables (applied when the greater trochanteric fragment was reattached). Kuroda et al. [4] also reported that the incidence of greater trochanteric complications was 5.0% (3/60 hips) in their THA cases via the modified Mostardi approach using two UHMPE fiber cables. In the present study, there were only a few greater trochanteric shifting cases (type C; 6.3%; 6/95 hips). The incidence of greater trochanteric fractures (type F) was 11.6% (11/95 hips) and almost identical to previous reports (Table 3). When the anterior and posterior knot outcomes were compared with previous findings, the outcome of the anterior knot was worse than past reports [3, 4]; however, the outcome of the posterior knot was identical to that reported by Oe et al. [3], although the patients were older and had femoral neck fracture. As the present study included patients with displaced femoral neck fracture and they were older, bone weakness was expected; thus, the incidence of greater trochanteric complications (greater trochanteric fracture in particular) were expected to be high. However, the result was equal to that of THA cases; thus, there was a confounding factor other than bone density.

**Table 3:** The ratio of the greater trochanteric complications

Parameters	Present study	Present study	Present study	Oe et al.	Kuroda et al.
	Whole group	Anterior group	Posterior group		
Mean age at opertion; years old	82.5 ± 7.4	82.4 ± 7.0	82.6 ± 7.9	64 ± uncertain	59.4 ± 12.2
Number of hips	95	47	48	466	60
Surgical form	BHA	BHA	BHA	THA	THA
Approach	Modified Dall	Modified Dall	Modified Dall	Modified Dall	Modified Mostardi
Knot position	Anterior+Posterior	Anterior	Posterior	uncertain	uncertain
All greater trochanteric complications	17.9%	27.6%	8.3%	7.9%	5.0%
Migration of the fragment	6.3%	10.6%	2.1%	uncertain	uncertain
Fracture of the greater trochanter	11.6%	17.0%	6.3%	uncertain	uncertain

Statistically, the age at operation, sex, size of the greater trochanteric fragment, type of the stem, and experience of the surgeon did not influence the greater trochanteric complications in the present study. In their THA cases performed via the modified Dall approach, Oe et al. [3] reported that the patients’ age and experience of the surgeon were risk factors for the complications of the greater trochanter. In the present study, the BMI and knot position were the risk factors (Table 2).

The incidence of greater trochanteric complications was significantly lower in the posterior group than in the anterior group. To our knowledge, no other institutions reported the difference in the knot position of the greater trochanteric fragment.

Although we could not examine such herein, the activity of daily living and strength of the gluteus medius muscle before the injury may influence the trochanteric complications; further examinations are necessary for THA cases.

**Fig. 5:**
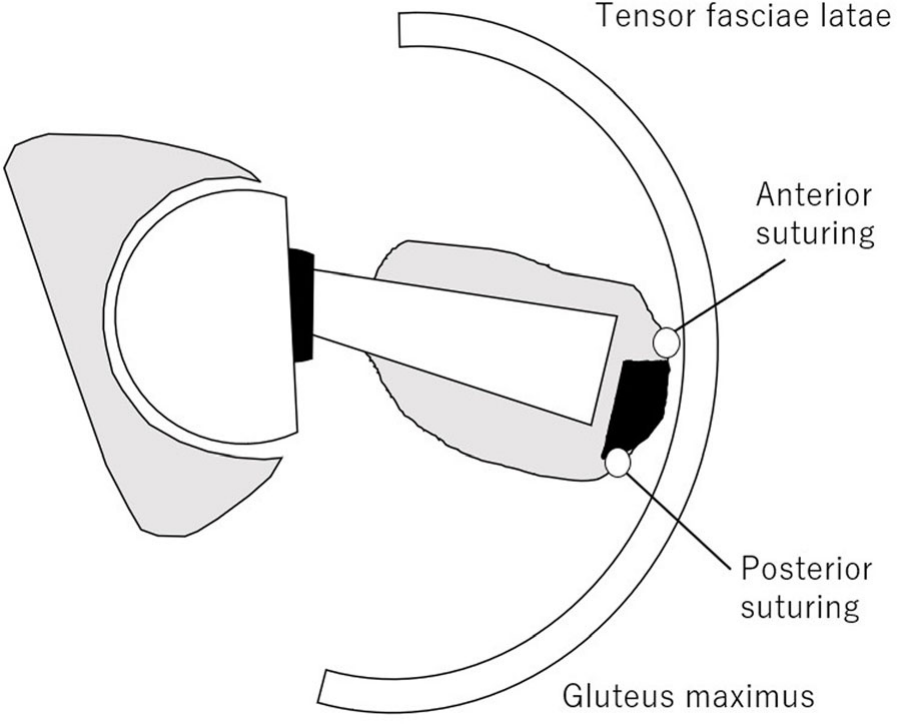
Relationship between the knot positions and muscles; the schema of the right hip is reflected from the top

The gluteus medius muscle can pull the fragment of the greater trochanter forward horizontally to the osteotomy surface; however, it is thought that the fragment digs into the ramp on the surface, which prevents the fragment from shifting anteriorly. When the knot was anteriorly positioned, the contact pressure with the tensor fascia lata muscle increased during external rotation, and it was thought that patients felt pain or discomfort and tended to assume the internal rotation position to avoid stimulation (Fig. 5). When the thighs assume the internal rotation position, the gluteus medius and gluteus minimus muscles contract and therefore pull the fragment horizontally to the osteotomy surface, causing the fragment to shift anteriorly. In addition, as the external rotation muscles become hyper-tense, the power to pull backwards increases, resulting in bone fracture at the non-cut area of the greater trochanter. Tip fracture occurs because the piriformis muscle and external rotator muscles, which lack plasticity, pull the tip of the greater trochanter strongly when the thighs assume the internal rotation position.

The limitations of the present study was that it was not a randomized study, it included relatively few cases, the follow-up period was short, it lacked data on clinical scores and functional outcomes, and the SC stem and Exeter stem were inserted depending on the surgeon’s discretion.

## Conclusion

The posterior knot positioning demonstrated better results than the anterior one. The present study findings are meaningful in that they may help improve the outcomes of the Dall approach. 

## Data Availability

The datasets analyzed during the study are available from the corresponding author upon reasonable request.

## Abbreviations

LAs: Lateral approaches
PAs: Posterior approaches
AAs: Anterior approaches
BHA: Bipolar hemiarthroplasty
THA: Total hip arthroplasty
UHMWPE: Ultra-high molecular weight polyethylene
BMI: Body mass index
